# Treatment of Solar Lentigines: A Systematic Review of Clinical Trials

**DOI:** 10.1111/jocd.70133

**Published:** 2025-03-27

**Authors:** Ghazal Mardani, Mohammad Javad Nasiri, Nastaran Namazi, Mehdi Farshchian, Fahimeh Abdollahimajd

**Affiliations:** ^1^ Department of Dermatology, Shohada‐e Tajrish Hospital Shahid Beheshti University of Medical Sciences Tehran Iran; ^2^ Skin Research Center Shahid Beheshti University of Medical Sciences Tehran Iran; ^3^ Department of Microbiology, School of Medicine Shahid Beheshti University of Medical Sciences Tehran Iran; ^4^ Department of Dermatology, David Geffen School of Medicine University of California Los Angeles Los Angeles California USA; ^5^ Clinical Research Development Unit, Shohada‐e Tajrish Hospital Shahid Beheshti University of Medical Sciences Tehran Iran

**Keywords:** laser, lentigo, solar lentigo, topical therapy, treatment

## Abstract

**Background:**

Solar lentigines, resulting from chronic UV exposure, are early signs of photoaging and can significantly affect individuals.

**Aims:**

This systematic review evaluates the efficacy, safety, and tolerability of treatments for solar lentigines in light of a lack of conclusive evidence regarding optimal therapy options.

**Methods:**

A systematic search of PubMed/Medline, EMBASE, Cochrane Library, and clinicaltrials.gov was conducted to identify relevant clinical trials published up to December 7, 2023. Inclusion criteria encompassed studies with patients diagnosed with solar lentigines, employing clinical trial methodologies and reporting clinical outcomes. Study quality was assessed using the Cochrane tool.

**Results:**

Forty‐one clinical trials involving 3234 patients aged 24–92 years were included. The most common effective topical treatment was a combination of mequinol 2% and tretinoin 0.01%, achieving efficacy rates between 52.6% and over 80%, particularly for facial lesions. Laser therapies demonstrated promising results: pulsed dye laser (27%–57% success), intense pulsed light (74.6%–90%), Q‐Switched laser (36.36%–76.6%), picosecond laser (67.9%–93.02%), and fractional CO_2_ laser (8%–23%). Cryotherapy yielded success in 37%–71.4%, while chemical peels with trichloroacetic acid achieved 12%–46%. Most adverse events were mild and transient, with local irritation from topical agents and mild pain from therapies being common. Pulsed dye and intense pulsed light lasers were less associated with post‐inflammatory hyperpigmentation, whereas cryotherapy was linked to more severe side effects.

**Conclusions:**

Laser therapy appears more effective than other modalities, with an acceptable safety profile. Combining lasers with specific topical agents may further improve outcomes and reduce PIH. However, additional large‐scale randomized trials are required to confirm these findings.

## 1 Introduction

Solar lentigines, also known as senile lentigines, are characterized by benign macular hyperpigmented lesions that vary in size and color [1]. These lesions are a result of localized proliferation of melanocytes and increases in melanin production within the epidermis, often in response to chronic UV light exposure [2]. In this way, solar lentigines lesions commonly appear on sun‐exposed areas of the skin, such as the hands, face, neck, and arms, as well as in populations with high levels of lifetime sun exposure [3, 4]. Although solar lentigines can emerge at any age, their prevalence increases with age, affecting more than 90% of individuals older than 50 years, particularly those with fair skin [5]. The development of solar lentigines is also influenced by genetic factors, with certain skin types and pigmentation levels predisposing individuals to a higher risk of developing these lesions [6, 7].

These lesions are not only a cosmetic concern but also represent early signs of photoaging and an indicator of cutaneous cancers [8, 9]. With the increasing focus on skin aesthetics and the growing aging population, the demand for effective treatment options for solar lentigines has escalated in recent years [9, 10]. A variety of treatment modalities have been developed to address the management of solar lentigines, reflecting the variable clinical presentations and individual patient preferences. These modalities range from topical medications to physical interventions [11, 12]. Physical therapy options include cryotherapy, chemical peels, laser therapy, and energy‐based devices. These modalities have shown promising clinical success rates. However, the potential side effects, inconsistent cosmetic outcomes, and the recurrence rates associated with these therapies need to be carefully weighed [13, 14, 15]. On the other hand, a variety of topical therapies with active compounds are employed, either alone or in combination with each other, which have been widely used for the management of solar lentigines. While these agents are convenient and non‐invasive, concerns regarding long‐term safety and the potential adverse effects have limited their utility in certain patient populations [16, 17, 18]. Additionally, the combination of physical interventions and topical agents has been applied for the treatment of solar lentigines [19].

The efficacy and safety profiles of the various therapeutic options for solar lentigines have been evaluated in several studies. However, there is a diversity in methodology and outcomes. In 2006, cryotherapy was recommended as the first‐line therapy for solar lentigines, based on the assessments of the Pigmentary Disorders Academy consensus [12]. However, a recent systematic review suggested that combination‐based and laser‐based treatments were the most efficacious therapeutic options [20]. Hence, there is a lack of treatment guidelines or an updated consensus regarding the optimal approach to managing solar lentigines. Therefore, the present systematic review on clinical trials aims to assess the current evidence on the efficacy, safety, and tolerability of various treatments for solar lentigines, which could provide evidence‐based guidance on clinical decision‐making for the treatment of solar lentigines.

## 2 Methods

### 2.1 Search Strategy

A systematic literature search using PubMed/Medline, EMBASE, Cochrane Library, and clinicaltrials.gov was conducted to identify studies investigating treatment modalities for solar lentigines, published up to December 7, 2023. The following keywords and MeSH terms were used: (“solar lentigo” or “solar lentigines” or “solar lentig*”, or “senile lentigo” or “senile lentigines” or “senile lentig*”) and (“treatment” or “therapy” or “clinical trial”). Only clinical trials written in English were included in the review. Additionally, backward and forward citation searches were conducted. The study adhered to the guidelines outlined in the Preferred Reporting Items for Systematic Reviews and Meta‐Analyses (PRISMA) 2020 statement [21]. The study protocol and detailed search strategy were registered in PROSPERO (CRD42024574497).

### 2.2 Study Selection

Regarding study selection, retrieved records were de‐duplicated using EndNote X9 software (Thomson Reuters, Toronto, ON, Canada). Two independent reviewers screened the records based on title/abstract first, followed by full‐text criteria to ensure alignment with the study objectives. Included studies met the following criteria: (1) patients were diagnosed with solar lentigines based on histological or clinical findings, (2) studies utilized clinical trial methodology, (3) studies included at least one therapeutic intervention, (4) studies reported clinical outcomes, and (5) studies were conducted on human patients.

Articles other than clinical trials, letters to the editors and animal studies, and articles in languages other than English, as well as studies for which the full text was not available, were excluded from the review.

### 2.3 Data Extraction

Data extraction involved the design of a data extraction form by two reviewers (G.M. and M.J.N.), who extracted pertinent information from eligible studies, resolving discrepancies through consensus and a third reviewer. Extracted data details included the following: (1) study characteristics (first author's name, study design, publication year, country, and sample size); (2) demographic information of patients (age and gender); (3) lentigines characteristics; (4) treatment protocol information (type and frequency/duration); and (5) follow‐up time; and (6) outcomes (outcome measured, main findings, and adverse effects).

### 2.4 Quality Assessment

Quality assessment of the studies was conducted by two reviewers utilizing the Cochrane tool for experimental (with comparison group) and semi‐experimental (without comparison group) studies [22]. A third reviewer was involved in case of inconsistencies. The Cochrane tool assesses studies based on the following criteria: (1) use of random sequence generation, (2) concealment of allocation to conditions, (3) blinding of participants and personnel, (4) blinding of outcome assessors, (5) completeness of outcome data and other factors, (6) selective reporting and other biases. Each study was categorized as follows: low risk of bias (no concerns regarding bias were identified), high risk of bias (concerns regarding bias were evident), or unclear risk of bias (information regarding bias was lacking or unclear).

## 3 Results

### 3.1 Basic Characteristics of Studies and Patients

**FIGURE 1:**
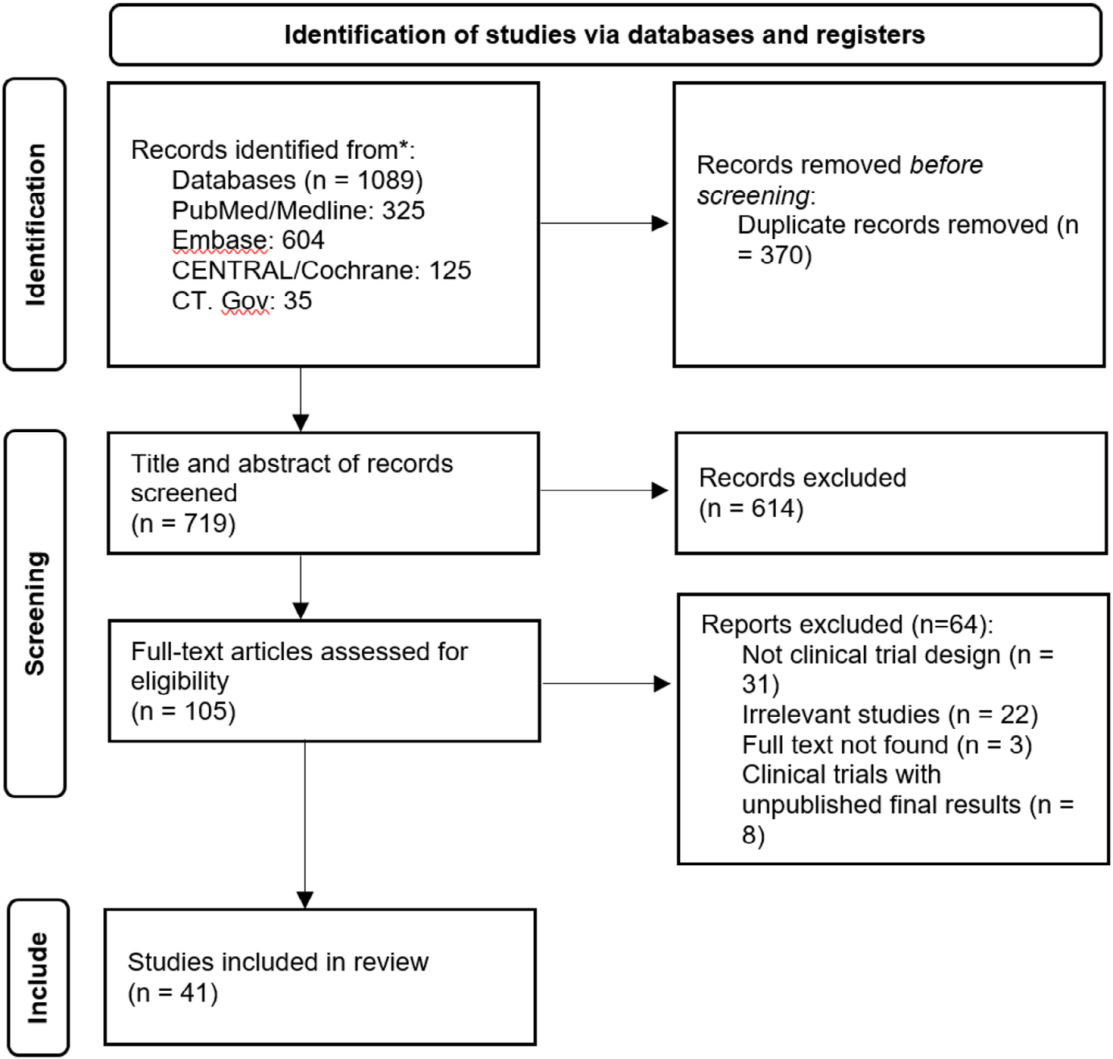
PRISMA flowchart.

Of the 1089 studies initially identified, subsequent to the elimination of duplicate records, the titles and abstracts of 719 studies were subjected to screening. Of these, 105 studies were selected for a full‐text evaluation. Ultimately, 41 studies met the inclusion criteria and were included in this systematic review [1, 13, 16, 17, 18, 19, 23, 24, 25, 26, 27, 28, 29, 30, 31, 32, 33, 34, 35, 36, 37, 38, 39, 40, 41, 42, 43, 44, 45, 46, 47, 48, 49, 50, 51, 52, 53, 54, 55, 56, 57] (Figure 1). Among the included studies, 16 were conducted in Asia, 10 in Europe, 11 in North America, 1 in South America, and 1 in Africa. Most of the studies (*n* = 32) were clinical trials with a comparison group (comparing two or more therapeutic modalities or with control/placebo), known as experimental studies, while the remaining studies were clinical trials without a comparison group, known as semi‐experimental studies. A total of 3234 patients were included in the studies, with ages ranging from 24 to 92 years. In 38 studies that reported the gender of the patients, 85.1% (*n* = 2703 out of 3176 participants) were female (Table 1).

**TABLE 1:** Characteristics of the studies included in the systematic review.

Author/year	Country	Study type	Number of samples	Age (year)	Gender (F/M)	Population characteristics	Intervention/comparison	Frequency/duration	Follow‐up
Topical treatments									
Makino et al. 2023 [1]	USA	Semi experimental	22	25–64	22/0	Subjects with FSP skin types I–IV and at least 1 age spot/solar SLs ≥ 3 mm on the face	Even & Correct Dark Spot Cream, SkinMedica	Once daily for 12 weeks	2, 4, 8, 12 weeks
Kim et al. 2021 [2]	Korea	Experimental	30	57.73 ± 2.70	22/8	Subjects with FSP skin types III–IV, diagnosed by a dermatologist as having facial SLs	EGF‐containing ointment after laser treatment vs. vehicle alone (petrolatum)	Twice daily for 4 weeks after laser treatment	4, 8 weeks
Ishikawa et al. 2019 [3]	Japan	Experimental	27	—	27/0	Subjects with clinically diagnosed SLs	Whitening lotion of L‐ascorbate‐2‐phosphate trisodium salt vs. control	Twice daily for 24 weeks	24 weeks
Arginelli et al. 2019 [4]	France	Experimental	36	50–79	34/2	Subjects with FSP skin type II–IV and diagnosis of SLs, at least 5 lesions on the surface of each hand	D‐Pigment, laboratories Eau Thermale, Avène vs. control	Once daily for 12 months	12, 24, 3, 48 weeks
Jiang et al. 2018 [5]	USA	Experimental	25	36–65	25/0	Subjects with FSP skin type II–IV and moderate to severe melasma and presence of SLs	Trifecting Night Cream, Envy Medical vs. control	Once daily, 2–3 times per week for the first 2 weeks, then once daily at night	4, 8, 16, 24 weeks
Campanati et al. 2016 [6]	Italy	Experimental	72	29–80	49/23	Subjects with FSP skin type II—IV and facial SLs	Application of pidobenzone 4% before and after Fractional CO_2_ laser or cryotherapy vs. control	Once daily for 2 weeks before and 10 weeks after the ablative treatment	12 weeks
Morag et al. 2015 [7]	Poland	Experimental	52	26–55	52/0	Subjects with hyperpigmentation, clinically and dermatoscopically diagnosed as SLs	Topical cream with the aqueous extract from leaf of five‐leaf serratula vs. control	Twice daily for 8 weeks	1, 4, 8 weeks
Hexsel D et al. 2015 [8]	Brazil	Experimental	50	57.5 ± 5	47/3	Subjects with FSP skin type II—IV and with at least five SLs in each hand dorsum, with at least 3 mm of diameter	TC cream (Tri‐Luma, Galderma, vs. control	Once daily for 2 weeks before cryotherapy and for 8 weeks, after cryotherapy	3, 7, 11 weeks (post cryotherapy)
Khemis et al. 2011 [9]	France	Experimental	30	49.1–80.8	27/3	FSP skin type II–IV and two or more SLs of ≥ 3 mm diameter	Serum‐containing L‐ascorbic acid 10% plus phytic acid 2% vs. control	Twice daily for 3 months	4, 8, 12, 16, 20 weeks
Katoulis et al. 2010 [10]	Greece	Experimental	30	47–75	28/2	Subjects with multiple SLs on hands	Undecylenoyl phenylalanine 2% in a cream vehicle vs. control	Twice daily for 12 weeks	4, 8, 12 weeks
Jarratt et al. 2006 [11]	USA	Experimental	216	35.2–84.4	183/33	Subjects with clinically confirmed SLs affecting the forearm and face	Mequinol/tretinoin vs. hydroquinone, vs. mequinol vs. tretinoin vs. control	Twice daily for 16 weeks	Until 40 weeks at regular intervals
Draelos et al. 2006 [12]	USA and Canada	Semi experimental	259	31–82	214/45	Subjects with FSP skin types II–V with ≥ 10 SL on the dorsal forearms/hands and ≥ 3 on the face	Mequinol/tretinoin	Twice daily for up to 24 weeks	12, 24 weeks
Ortonne et al. 2004 [13]	France and Belgium	Semi experimental	406	62.3 ± 9.3	349/57	Subjects with FSP skin type I–V and clinically diagnosed SLs on forearms/hands and the face	Mequinol/tretinoin	Twice daily for up to 24 weeks	4 weeks (post treatment)
Kang et al. 2003 [14]	USA	Experimental	90	63.1	69/21	Subjects with diagnosed actinic keratoses and SLs lesions with at least 5 mm in diameter	Adapalene 0.1% vs. adapalene 0.3% vs. control	Once daily for 4 weeks, followed by twice‐daily up to 9 months, if tolerated	12, 36, 36 weeks
Hermanns et al. 2002 [15]	Belgium	Experimental	30 20	42–57 51–56	30/0 20/0	Study 1: subjects with SLs lesions on the dorsal forearms and back of the hands Study 2: menopausal women with SLs on the dorsum of the hands	Study 1: Stabilized soy extract vs. control Study 2: Melanex duo, Paraphar vs. Skinoren, Schering	Study 1: twice daily for 2 months Study 2: twice daily for 3 months	Study 1: 8 weeks Study 8, 12 weeks
Fleischer et al. 2000 [16]	USA	Experimental	595 580	34–85 33–90	486/109 482/98	Subjects with clinically diagnosed SLs on their forearms, backs of hands and face	Mequinol/tretinoin vs. tretinoin vs. mequinol vs. control	Twice daily for 24 weeks	4, 24 weeks
Physical treatments									
Abd. El‐Naby et al. 2022 [17]	Egypt	Experimental	22	48–75	21/1	Subjects with FSP skin types III–IV and pathologically confirmed SLs	One stacked PDL session vs. two stacked PDL sessions	Group I: one session Group II: two sessions at a one‐month interval	24 weeks
Kim et al. 2020 [18]	Korea	Experimental	20	27–72	20/0	Subjects with FSP skin types III–V and clinically obvious SLs on both sides of the face	532 nm PS laser vs. 532 nm QS Nd:YAG laser treatment	One session	2, 4, 8, 12 weeks
Dawood et al. 2020 [19]	Pakistan	Experimental	120	20–65	88/32	Subjects with FSP skin type III–IV and clinically diagnosed SLs	Cryotherapy (liquid nitrogen) vs. 534 nm QS Nd: YAG	8 sessions at 15 days' intervals	16 weeks
Friedmann et al. 2019 [20]	USA	Semi experimental	16	41–79	15/1	Subjects with FSP skin type I–III and resistant, age or sun‐related flat benign pigmentation, on face or hands	IPL with KTP lasers	3 sessions at a 1‐month intervals	4, 12, 24 weeks (post the last session)
Vachiramon et al. 2018 [21]	Thailand	Experimental	28	61.7 ± 6.9	29/1	Subjects with at least 2 clinically diagnosed SLs on the upper extremities	Double frequency 532‐nm Nd:YAG laser vs. double frequency 532‐nm Nd:YAG ps laser	A single session	6, 12 weeks
Negishi et al. 2018 [22]	Japan	Semi experimental	20	53.7 ± 9.75	20/0	Subjects with clinically diagnosed facial SLs larger than 6 mm in diameter FSP skin type II‐IV	Double frequency 532‐nm Qs Nd:YAG ns laser vs. double frequency 532‐nm Nd:YAG ps laser	A single session	4, 12 weeks
Bohnert et al. 2018 [23]	USA	Experimental	10	40–63	10/0	Subjects with FSP skin type I‐III and mild‐to‐severe SLs	Single‐pulsed 1064‐nm Nd:YAG with dual‐pulsed 532‐nm/1064‐nm QS laser	Up to 6 sessions at 2–3 weeks intervals	4 weeks (post the last session)
Kaminaka et al. 2017 [24]	Japan	Experimental	8	39–61	8/0	Subjects with FSP skin type III‐IV and clinically and histopathologically confirmed pigmented SLs on both cheeks	Low‐Fluence 1064‐nm Qs Nd:YAGLaser vs. control	10 sessions at 1‐week interval	4 weeks (post 5th and 10th session), 12, 24 weeks
Vachiramon et al. 2016 [25]	Thailand	Experimental	25	64.5 ± 9.5	24/1	Subjects with FSP skin type III‐IV and at least two diagnosed lesions of SLs on the upper extremities.	532‐ nm Qs Nd:YAG vs. fractional CO_2_ laser	A single session	6, 12 weeks
Imhof et al. 2016 [26]	Switzerland	Experimental	15	57–70	14/1	Subjects with FSP skin type III‐IV and symmetrically distributed diagnosed lesions of SLs on the back of both hands	QS Ruby laser vs. hydroquinone/tretinoin/dexamethasone	Group I:1 or 2 session at 4 weeks' interval Group II: once daily for 7 weeks	4, 8, 20 weeks
Schoenewolf et al. 2015 [27]	Switzerland	Experimental	11	48–70	10/1	Subjects with clinically and dermatoscopically diagnosed SLs symmetrically localized on both dorsal hands	Qs Ruby laser vs. Fractional CO_2_ laser	Three sessions at 0, 4, 8 weeks	16, 24 weeks
Noh et al. 2015 [28]	Korea	Experimental	8	42–60	8/0	Subjects with FSP skin types III–IV and clinically diagnosed facial SLs	660‐nm Qs Nd:YAG vs. 532‐nm Qs Nd:YAG Laser	A single session	4, 8 weeks
Jun et al. 2014 [29]	Korea	Experimental	15	24–92	11/4	Subjects with FSP skin types III–V and light facial SLs	532‐nm Qs Nd:YAG laser vs. Er:YAG micropeel	A single session	2, 4 weeks
Jun et al. 2013 [30]	Korea	Experimental	15	30–64	14/1	Subjects with light facial SLs and FSP skin types III–V	532‐nm QS Nd:YAG ns and Er:YAG micropeel vs. QS Nd:YAG alone	A single session	2, 4 weeks
Ghaninejhadi et al. 2013 [31]	Iran	Semi experimental	21	39–71	18/3	Subjects with FSP skin type II–IV and histopathologically confirmed SLs on the face or hands	PDL therapy	A single session	24 months
Seirafi et al. 2011 [32]	Iran	Experimental	22	28–67	20/2	Subjects with FSP skin types II–V and SLs on face or hands based on clinical diagnose	Cryotherapy (liquid nitrogen) vs. 595‐nm PDL with compression	A single session	4 weeks
Sasaya et al. 2011 [33]	Japan	Semi experimental	31	40–74	31/0	Subjects with SLs on the back of hands	IPL therapy a 515‐nm cutoff filter	3–5 sessions at 3–4 weeks interval	4 weeks (post last session)
Golforoushan et al. 2010 [34]	Iran	Experimental	30	38–64	27/3	Subjects with FSP I–III and clinical diagnosis of SLs	Cryotherapy (liquid nitrogen) vs. 35% TCA solution	2 sessions at 1 month interval	8 weeks
Sadighha et al. 2008 [35]	Iran	Semi experimental	91	38–64	68/21	Subjects with FSP skin type II–IV and clinical diagnosis of SLs	694‐nm Qs Ruby Laser	One or two single sessions at 1 month interval	4, 24 weeks
Raziee et al. 2008 [36]	Iran	Experimental	25	30–71	25/0	Subjects with FSP skin type II–IV, and at least five lesions on each hand based on clinical diagnosis	TCA 33% solution vs. cryotherapy (liquid nitrogen)	A single session	8 weeks
Lugo‐Janer et al. 2003 [37]	Puerto Rico	Experimental	25	—	25/0	Subjects with SLs on the dorsal of each hand	30% TCA solution vs. cryosurgery (liquid nitrogen)	A single session	8 weeks
Todd et al. 2000 [38]	USA	Experimental	27	—	—	Subjects with at least 6 easily identifiable SLs on the back of hand	Cryotherapy vs. the Medlite II frequency‐doubled Qs Nd:YAG laser vs. the HGM K1 krypton laser vs. the DioLite a532‐nm diode‐pumped vanadate laser	A single session	12 weeks
Bjerring et al. 2000 [39]	Denmark	Semi experimental	18	—	—	Subjects with diagnosis of SLs	IPL therapy	A single session	8 weeks
Hexsel et al. 2000 [40]	Brazil	Experimental	58	31–96	58/0	Subjects with clinally diagnosed SLs and FSP skin type of I–V	Cryotherapy (liquid nitrogen) vs. local dermabrasion	A single session	1 day 1, 2, 3, 24 weeks
Stern et al. 1994 [41]	USA	Experimental	13	—	—	Subjects with diagnosis of SLs	Cryotherapy vs. argon laser light vs. low‐fluence CO_2_ laser irradiation	A single session	8 weeks

*Note:* Please refer to the Appendix S1 to view Tables' references.

Abbreviations: F, female; FSP, Fitzpatrick skin type; M, male; SL, solar lentigo; TCA, trichloroacetic acid.

### 3.2 Quality of the Included Studies

Based on the Cochrane tool, which was used to evaluate the quality of the clinical trials, 13 studies were double‐blinded, randomized trials and had a low risk of bias. Twelve and 27 studies had a high risk for assessor and participant blinding, respectively. Twelve studies had a high risk of bias for randomization and group concealment (Table 2).

**TABLE 2:** Quality assessment of the studies included in the systematic review (the Cochrane tool).

Authors	Random sequence generation	Allocation concealment	Blinding of participants and personnel	Blinding of outcome assessment	Incomplete outcome data	Selective reporting	Other bias
Makino et al.	High risk	High risk	High risk	High risk	Low risk	Low risk	Low risk
Kim et al.	Low risk	Low risk	Low risk	Low risk	Low risk	Low risk	Low risk
Ishikawa et al.	High risk	High risk	Low risk	Low risk	Low risk	Low risk	Low risk
Arginelli et al.	Low risk	Low risk	High risk	High risk	Low risk	Low risk	Low risk
Jiang et al.	Low risk	Low risk	Low risk	Low risk	Low risk	Low risk	Low risk
Campanati et al.	Low risk	Low risk	High risk	High risk	Low risk	Low risk	Low risk
Morag et al.	Low risk	Low risk	Low risk	Low risk	Low risk	Low risk	Low risk
Hexsel et al.	Low risk	Low risk	High risk	Low risk	Low risk	Low risk	Low risk
Khemis et al.	Low risk	Low risk	Low risk	Low risk	Low risk	Low risk	Low risk
Katoulis et al.	Low risk	Low risk	Low risk	Low risk	Low risk	Low risk	Low risk
Raziee et al.	Low risk	Low risk	High risk	Low risk	Low risk	Low risk	Low risk
Jarratt et al.	Low risk	Low risk	Low risk	Low risk	Low risk	Low risk	Low risk
Draelos et al.	High risk	High risk	High risk	High risk	Low risk	Low risk	Low risk
Ortonne et al.	High risk	High risk	High risk	High risk	Low risk	Low risk	Low risk
Lugo‐Janer et al.	Low risk	Low risk	High risk	Low risk	Low risk	Low risk	Low risk
Kang et al.	Low risk	Low risk	High risk	Low risk	Low risk	Low risk	Low risk
Hermanns et al.	Low risk	Low risk	High risk	High risk	Low risk	Low risk	Low risk
Hexsel et al.	Low risk	Low risk	High risk	Low risk	Low risk	Low risk	Low risk
Fleischer et al.	Low risk	Low risk	Low risk	Low risk	Low risk	Low risk	Low risk
Abd. El‐Naby et al.	Low risk	Low risk	Low risk	Low risk	Low risk	Low risk	Low risk
Kim et al.	Low risk	Low risk	High risk	Low risk	Low risk	Low risk	Low risk
Dawood et al.	Low risk	Low risk	High risk	High risk	Low risk	Low risk	Low risk
Friedmann et al.	High risk	High risk	High risk	High risk	Low risk	Low risk	Low risk
Vachiramon et al. (2018)	Low risk	Low risk	Low risk	Low risk	Low risk	Low risk	Low risk
Negishi et al.	High risk	High risk	High risk	Low risk	Low risk	Low risk	Low risk
Bohnert et al.	Low risk	Low risk	Low risk	Low risk	Low risk	Low risk	Low risk
Kaminaka et al.	Low risk	Low risk	Low risk	Low risk	Low risk	Low risk	Low risk
Vachiramon et al. (2016)	Low risk	Low risk	High risk	Low risk	Low risk	Low risk	Low risk
Imhof et al.	High risk	High risk	High risk	Low risk	Low risk	Low risk	Low risk
Schoenewolf et al.	Low risk	Low risk	High risk	Low risk	Low risk	Low risk	Low risk
Noh et al.	Low risk	Low risk	Low risk	Low risk	Low risk	Low risk	Low risk
Jun et al. (2014)	Low risk	Low risk	High risk	Low risk	Low risk	Low risk	Low risk
Jun et al. (2013)	Low risk	Low risk	High risk	Low risk	Low risk	Low risk	Low risk
Ghaninejhadi et al.	High risk	High risk	High risk	High risk	Low risk	Low risk	Low risk
Seirafi et al.	Low risk	Low risk	High risk	Low risk	Low risk	Low risk	Low risk
Sasaya et al.	High risk	High risk	High risk	High risk	Low risk	Low risk	Low risk
Golforoushan et al.	Low risk	Low risk	High risk	Low risk	Low risk	Low risk	Low risk
Sadighha et al.	High risk	High risk	High risk	Low risk	Low risk	Low risk	Low risk
Todd et al.	Low risk	Low risk	High risk	Low risk	Low risk	Low risk	Low risk
Bjerring et al.	High risk	High risk	High risk	High risk	Low risk	Low risk	Low risk
Stern et al.	Low risk	Low risk	High risk	High risk	Low risk	Low risk	Low risk

### 3.3 Topical Treatment With Active Compounds

Among the included studies, 16 evaluated the efficacy and safety of topical treatments with active compounds in treating solar lentigines. Five studies focused on patients with solar lentigines on the face [23, 25, 28, 33, 38], while six studies included patients with solar lentigines on both the upper extremities and the face [5, 17, 18, 50, 51, 54]. In 5 studies, the lesions were located on the upper limbs (including the dorsum of hands, and forearms) [16, 30, 41, 46, 55]. The duration of treatment varied from 7 weeks to 12 months. The active compounds used in the treatment of solar lentigines varied across the studies. The topical treatment combining 4‐hydroxyanisole (mequinol) 2% and tretinoin 0.01% has undergone extensive evaluation in various studies (Table 3). The topical combination product of mequinol 2% and tretinoin 0.01% was found to be associated with clinical success rates of 52.6% [17], 56.0% [50], 68.0% [51], and over 75% [52] for lesions on the upper limbs, while 56.3% [17], > 70% [50], 76.4% [51], and > 80% [52] for lesions on the face. Treatment with undecylenoyl phenylalanine 2% for solar lentigines on the hands resulted in 63.3% moderate and 36.6% marked improvement, with 80% of patients reporting increased satisfaction [46]. Topical adapalene gel 0.1% and 0.3% were effective in lightening solar lentigines lesions in 57% and 59% of patients, respectively [54]. Another combination topical therapy with hydroquinone 5%, tretinoin 0.03%, and dexamethasone 0.03% was about 50% effective in decreasing solar lentigines lesions [37]. Treatment with Dark Spot Cream by SkinMedica was found to improve hyperpigmentation and skin tone of lesions by about 15% and lesion size on the face, with 85% of patients reporting enhanced satisfaction [23]. Topical lotions incorporating ascorbate derivatives, including 6% L‐ascorbate‐2‐phosphate trisodium salt [28] and L‐ascorbic acid 10% and phytic acid 2% [18] demonstrated improvements in mean scores of skin brightness when compared to a control group. Moreover, a topical agent containing 0.5% phenylethyl resorcinol, 0.05% retinaldehyde, and 0.1% tocopheryl glucoside proved effective in treating solar lentigines lesions, leading to a significant reduction in hyperpigmentation, improvements in clinical and histological features, and increased patient satisfaction compared to controls [30]. Also, Trifecting Night Cream 1.0 showed global improvements in solar lentigines [33]. A plant extract cream containing 2.51% arbutin successfully lightened skin and decreased melanin levels in 56% of patients with solar lentigines in comparison to controls [41]. Additionally, a stabilized soy extract demonstrated a modest yet superior lightening effect when compared to a 20% azelaic acid formulation and another formulation with 5% ascorbyl glucosamine, 1% kojic acid, and α‐hydroxy acid esters, which showed ineffectiveness for solar lentigines [16] (Table 3).

**TABLE 3:** Main results.

Author/year	Intervention	Outcomes measured	Main findings	Adverse effects reported
Topical treatments				
Makino et al. 2023 [1]	Even & Correct Dark Spot Cream, SkinMedica	– Clinical assessment for pigmentation and size reduction – Participant self‐assessments – Tolerability assessment – Adverse effects	Significant improvement and high participant satisfaction	– No serious adverse effects – Erythema, dryness, and peeling were observed – Well tolerated
Kim et al. 2021 [2]	EGF‐containing ointment after laser treatment vs. vehicle alone (petrolatum)	– Clinical assessment for pigmentation reduction – Participant self‐assessments – Adverse effects	Synergistic therapeutic effect as adjuvant treatment with QS‐532 Nd:YAG laser	– No serious adverse effects
Ishikawa et al. 2019 [3]	Whitening lotion of L‐ascorbate‐2‐phosphate trisodium salt vs. control	– Clinical assessment for pigmentation reduction – Melanin index	Weak but significant anti‐pigmenting effect	—
Arginelli et al. 2019 [4]	D‐Pigment, laboratories Eau Thermale, Avène vs. control (Moisturizer)	– Clinical assessment for pigmentation reduction – Participant self‐assessments – Assessment of Tolerability – Histopathological assessment – Adverse events	More effective compared to a moisturizing product	– No serious adverse effects
Jiang et al. 2018 [5]	Trifecting Night Cream, Envy Medical vs. control	– Clinical assessment for pigmentation reduction – Participant self‐assessment – Tolerability assessment – Chroma Meter measurements	Effective for the treatment of moderate to severe melasma, SLs, and periocular lines and wrinkles	– No serious adverse effects – Mild erythema, itching, and dryness
Campanati et al. 2016 [6]	Application of pidobenzone 4% before and after Fractional CO_2_ laser or cryotherapy vs. control	– Clinical assessment for pigmentation reduction – Participant self‐assessment	The combination of cryotherapy and topical pidobenzone 4% has been found to be the most useful treatment	—
Morag et al. 2015 [7]	Topical cream with the aqueous extract from leaf of five‐leaf serratula vs. control	– Clinical assessment for pigmentation reduction – Adverse effects	Relatively effective and safe for lightening of SLs	– No serious adverse events
Hexsel et al. 2015 [8]	TC cream (Tri‐Luma, Galderma (containing 0.01% fluocinolone acetonide, 4% hydro‐quinone and 0.05% tretinoin) vs. control	– Clinical assessment for pigmentation reduction – Mean decrease in number of lesions – Participant self‐ satisfaction – Adverse effects	Effective and safe to enhance the resolution of SLs	– No serious adverse events ˗ Erythema
Khemis et al. 2011 [9]	Serum‐containing L‐ascorbic acid 10% plus phytic acid 2% vs. control	– Clinical assessment for pigmentation reduction – Adverse effects	Significantly efficacious and well tolerated	– Transitory mild‐to‐moderate local irritation – Depigmentation – Tingling
Katoulis et al. 2010 [10]	Undecylenoyl phenylalanine 2% in a cream vehicle vs. control	– Clinical assessment for pigmentation reduction – Decrease in number of lesions – Patient self‐assessment – Adverse effects	Effective and well‐tolerable	– Erythema – Itching – Burning sensation
Jarratt et al. 2006 [11]	Mequinol/tretinoin vs. hydroquinone, vs. mequinol vs. tretinoin vs. control	– Clinical assessment for pigmentation reduction – The time to reach a response – Tolerability assessment – Adverse events	Mequinol/tretinoin solution is a highly effective and well‐tolerated, being superior to hydroquinone	– No serious adverse effects – Burning – Stinging – Tingling – Pruritus – Rash – Erythema
Draelos et al. 2006 [12]	Mequinol/tretinoin	– Clinical assessment for pigmentation reduction – The time to reach a response – Adverse effects	Effective and safe treatment in ethnic populations, and in those with dark skin types	– Erythema – Skin discomfort – Halo‐hypopigmentation – Irritant dermatitis – Desquamation – Pruritus – Hypopigmentation – Dry skin
Ortonne et al. 2004 [13]	Mequinol/tretinoin	– Clinical assessment for pigmentation reduction Adverse effects	Effective, convenient, and safe	– No serious adverse events – Erythema – Burning – Stinging – Tingling – Skin irritation – Desquamation Pruritus – Dermatitis – Hypopigmentation
Kang et al. 2003 [14]	Adapalene 0.1% vs. adapalene 0.3% vs. control	– Clinical assessment for pigmentation reduction – Adverse effects	Both Adapalene gel 0.1% and 0.3% were well tolerated and improved the lesions	– No serious adverse events – Mild to moderate dermatitis
Hermanns et al. 2002 [15]	Study 1: Stabilized soy extract vs. control Study 2: Melanex duo, Paraphar vs. Skinoren, Schering	– Clinical assessment for pigmentation reduction – Melanex index – Skinoren index	A stabilized soy extract showed a better although modest lightening effect than placebo were ineffective Melanex duo, and Skinoren, were ineffective	—
Fleischer et al. 2000 [16]	Mequinol/tretinoin vs. tretinoin vs. mequinol vs. control	– Clinical assessment for pigmentation reduction – Participant self‐assessment – Adverse events	Mequinol/tretinoin is well tolerated and superior to either active component	– Erythema – Burning – Stinging – Tingling – Desquamation – Pruritus – Skin irritation – Halo hypopigmentation
Physical treatments				
Abd. El‐Naby et al. 2022 [17]	One stacked PDL session vs. two stacked PDL sessions	– Clinical assessment for pigmentation reduction – Histopathological evaluation – Decrease in number of lesions – Adverse effects	Significant better clinical and histopathological outcomes in both PDL techniques	– No serious adverse effects
Kim et al. 2020 [18]	532 nm PS laser vs. 532 nm QS Nd:YAG laser treatment	– Clinical assessment for pigmentation reduction – Participant self‐assessments – Tolerability assessment – Adverse effects	532 nm PS Nd:YAG laser seems to be more effective and safer than 532 nm QS‐Nd:YAG laser treatment	– No serious adverse effects
Dawood et al. 2020 [19]	Cryotherapy (liquid nitrogen) vs. 534 nm QS Nd: YAG	– Clinical assessment for pigmentation and size reduction – Decrease in number of lesions	Q‐switch Nd: YAG laser therapy is significantly superior to liquid nitrogen cryotherapy	—
Friedmann et al. 2019 [20]	IPL with KTP lasers	– Clinical assessment for pigmentation reduction – Participant self‐assessment – Melanin index – Adverse effects	Well‐tolerated and effective method	– Erythema – Hypopigmentation
Vachiramon et al. 2018 [21]	Double frequency 532‐nm Nd:YAG laser vs. double frequency 532‐nm Nd:YAG ps laser	– Clinical assessment for pigmentation reduction – Participant self‐assessments – Luminance score – Tolerability assessment – Adverse events	Qs 532‐nm Nd:YAG ns laser seems to be more cost‐effective	– No serious adverse effects – Hyperpigmentation (in KTP 532‐nm Ps laser treatment) – Hyperpigmentation (in both treatment) – Prolonged erythema (in Q‐switched KTP532‐nm ns Laser treatment)
Negishi et al. 2018 [22]	Double frequency 532‐nm Qs Nd:YAG ns laser vs. double Frequency 532‐nm Nd:YAG ps laser	– Clinical assessment for pigmentation reduction – Participant self‐assessment – Melanin index – Histolopathological asssessment – Adverse events	Dual‐wavelength and dual‐pulse width ps Nd:YAG laser can be a safer and more effective treatment over conventional treatments	– No serious adverse effects – Mild pain – Post‐inflammatory hyperpigmentation(PIH) – Hypopigmentation – Prolonged erythema
Bohnert et al. 2018 [23]	Single‐pulsed 1064‐nm Nd:YAG vs. dual‐pulsed 532‐nm/1064‐nm QS laser	– Clinical assessment – Participant self‐assessment – Adverse events – Tolerability assessment	Dual‐pulsed 532‐nm/1064‐nm Q‐switched laser treatment can result in superior and safe global improvement	– No serious adverse – Mild erythema and swelling in all patients immediately post treatment, but resolved within 24 h
Kaminaka et al. 2017 [24]	Low‐Fluence 1064‐nm Qs Nd:YAGLaser vs. control	– Clinical assessment for pigmentation and size reduction – Assessment the lesion recurrence – Melanin index – Participant self‐assessment – Histopathological assessment – Tolerability assessment – Adverse effects	Low‐fluence QS Nd:YAG is effective, safe, tolerable in treating small type SLs	– No systemic and serious adverse events – Slight, transient erythema, Swelling – Mild pain – PHI – Skin dryness – Pruritus
Vachiramon et al. 2016 [25]	532‐nm Qs Nd:YAG vs. fractional CO_2_ laser	– Clinical assessment for pigmentation reduction – Melanin index – Participant self‐assessment – Tolerability assessment – Adverse effects	Qs Nd:YAG is more effective than fractional CO_2_ laser but requires longer healing time and produces had more pain	– No serious adverse events – Mild pain immediately after laser – Mild post‐inflammatory hyperpigmentation
Imhof et al. 2016 [26]	QS Ruby laser vs. hydroquinone/tretinoin/dexamethasone	– Clinical assessment for pigmentation reduction – Adverse effects	– Both methods were effective and safe – QS Ruby laser provides faster, superior, and long‐lasting lightening effect	– No serious adverse
Schoenewolf et al. 2015 [27]	Qs Ruby laser vs. Fractional CO_2_ laser	– Clinical assessment for pigmentation reduction – Participant self‐assessment – Adverse effects	QS‐ruby laser was superior and more effective to the ablative CO_2_ fractional laser	– No serious adverse events – Discomfort during the Q‐switched ruby laser – Erythema immediately after treatment – Slight edema
Noh et al. 2015 [28]	660‐nm Qs Nd:YAG vs. 532‐nm Qs Nd:YAG Laser	– Clinical assessment for pigmentation reduction – Participant self‐assessment – Adverse effects	Both 660‐nm and 532‐nm QS Nd:YAG lasers were effective with high patient satisfaction	– No serious adverse events
Jun et al. 2014 [29]	532‐nm Qs Nd:YAG laser vs. Er:YAG micropeel	– Clinical assessment for pigmentation reduction – Participant self‐assessment – Adverse effects	The immediate effects were better with the Qs Nd:YAG laser but there was no great difference between the two laser types at 1‐month follow‐up	– No serious adverse events – PIH
Jun et al. 2013 [30]	532‐nm QS Nd:YAG ns and Er:YAG micropeel vs. QS Nd:YAG alone	– Clinical assessment for pigmentation reduction – Participant self‐assessment – Adverse effects	Nd:YAG laser alone is considered to have more favorable qualities than combined treatment	– No serious adverse events – PIH
Ghaninejhadi et al. 2013 [31]	PDL therapy	– Clinical assessment for pigmentation reduction – Participant self‐assessment – Adverse effects	PDL is a safe and effective therapeutic option	– No serious adverse events – Erythema – Mild burn – Transient PIH
Seirafi et al. 2011 [32]	Cryotherapy (liquid nitrogen) vs. 595‐nm PDL with compression	– Clinical assessment for pigmentation reduction – Adverse effects	PDL with compression is superior to cryotherapy in darker skin types	– No serious adverse events – PIH
Sasaya et al. 2011 [33]	IPL therapy a 515‐nm cutoff filter	– Clinical assessment for pigmentation reduction – Participant self‐assessment – Tolerability assessment – Adverse effects	Effective and well tolerated	– No adverse events
Golforoushan et al. 2010 [34]	Cryotherapy (liquid nitrogen) vs. 35% TCA solution	– Clinical assessment for pigmentation reduction	TCA solution had similar efficacy compared to cryotherapy but fewer side effects	– Blister – Hypopigmentation – Permanent scar – Severe itching – Sever pain (in cryotherapy treatment) – Erythema – Severe itching – Edema (in TCA solution treatment)
Sadighha et al. 2008 [35]	694‐nm Qs Ruby Laser	– Clinical assessment for pigmentation reduction – Adverse effects	Effective and safe procedure even in dark‐skinned individuals	– No serious adverse events
Raziee et al. 2008 [36]	TCA 33% solution vs. cryotherapy (liquid nitrogen)	– Clinical assessment for pigmentation reduction – Patient self‐assessment – Tolerability assessment – Adverse effects	Cryotherapy was more likely to produce substantial lightening of the SLs than TCA 33% solution but more painful and took more time to heal	– No serious adverse events – PIH
Lugo‐Janer et al. 2003 [37]	30% TCA solution vs. cryosurgery (liquid nitrogen)	– Clinical assessment for pigmentation reduction – Participant self‐assessment – Adverse effects	Cryotherapy was more likely to produce significant lightening effect than 30% TCA solution but was more painful and took longer to heal	– No serious adverse events – Pain
Todd et al. 2000 [38]	Cryotherapy vs. the Medlite II frequency‐doubled Qs Nd:YAG laser vs. the HGM K1 krypton Laser vs. the DioLite a532‐nm diode‐pumped vanadate laser	– Clinical assessment for pigmentation reduction – Adverse effects	Laser therapy is superior to cryotherapy Of the laser systems, the Medlite II frequency‐doubled Q‐switched Nd:YAG laser is the most effective	– No serious adverse events – Erythema – Pain
Bjerring et al. 2000 [39]	IPL therapy	– Clinical assessment for pigmentation reduction – Adverse effects	Found to be effective	– Erythema – Separation of the epidermis followed by crusting or ulceration and erythema – Pain – Pin‐prink feeling followed by a burning sensation
Hexsel et al. 2000 [40]	Cryotherapy (liquid nitrogen) vs. local dermabrasion	– Time required to perform procedure – Post‐surgery signs and symptoms – Tolerability assessment – Adverse effects	Localized dermabrasion is an efficacious and effective technique comparable to cryotherapy	– No serious adverse events
Stern et al. 1994 [41]	Cryotherapy vs. argon laser light vs. low‐fluence CO_2_ laser irradiation	– Clinical assessment for pigmentation reduction	Cryotherapy was superior to argon and CO_2_ laser therapy	—

*Note:* Please refer to the Appendix S1 to view Tables' references.

Besides, three studies investigated the use of topical medications as adjunct therapies to physical interventions [19, 25, 38]. Among these, the application of an ointment containing 1 μg/g recombinant human epidermal growth factor following QS 532 nm Nd:YAG laser treatment for solar lentigines resulted in a significantly greater reduction in the melanin index of lesions and a lower incidence of post‐inflammatory hyperpigmentation (7.14% vs. 37.5%) compared to the control group [25]. Treatment of solar lentigines lesions with topical pidobenzone 4% after ablative cryotherapy demonstrated marked attenuation in hyperpigmentation compared to baseline, as assessed both clinically and through self‐assessments [38]. Furthermore, the use of a topical cream containing 0.01% fluocinolone acetonide, 4% hydroquinone, and 0.05% tretinoin post‐cryotherapy significantly reduced melanin levels and lentigines count, enhancing the treatment outcomes of cryotherapy [19] (Table 3).

In the context of adverse effects, the majority of skin‐related adverse events were mild and transient, with topical agents generally well tolerated. Commonly reported adverse effects included local skin irritation, erythema, itching, peeling, dryness, burning sensation, stinging, and tingling, followed by hypopigmentation and/or halo hypopigmentation. Importantly, no serious or severe adverse events, such as scarring or atrophy, were reported in the majority of the studies (Table 3).

### 3.4 Physical Therapy

In a total of 25 studies, the efficacy and safety of various physical therapies were evaluated for the treatment of solar lentigines. In 21, 8, and 3 studies, multiple laser modalities, cryotherapy, and chemical peels were assessed, respectively. Of these studies, six concentrated on patients with solar lentigines located on the facial region [26, 32, 40, 42, 43], while 10 involved patients with lesions on the upper limbs (dorsum of the hands, forearms, and arms). Additionally, nine studies assessed patients with solar lentigines present on both the face and upper limbs. The frequency of treatments administered across these studies ranged from one to ten sessions, with the majority reporting a regimen of one or two sessions (Table 3). Among the patients who received one session of Pulsed dye laser (PDL) therapy, 27.3% [24] to 57% [44] showed marked/excellent improvement. High patient satisfaction (over 80%) and great reductions in both lesion number and pigmentation were found following PDL therapy [24, 44]. Importantly, PDL therapy had a greater lightening effect on solar lentigines lesions for skin types III and IV and fewer side effects than cryotherapy [13].

Treatment of facial and hand lesions with intense pulsed light (IPL) using a KTP filter resulted in significant improvements in pigmentation. After 1 month, 74.6% of treated facial areas and 90% of treated hand regions achieved good to excellent outcomes, with over 60% of patients maintaining these improvements at the 6‐month mark. Satisfaction rates and reduction in melanin index were also notable [29]. Additionally, IPL with a 515‐nm filter showed a significant efficacy in the treatment of solar lentigines, with > 50% and > 75% improvement in 62% and 23% of cases, with no occurrence of post‐inflammatory hyperpigmentation (PIH) [45]. Similarly, another study found that IPL treatment resulted in pigment reduction in 94.4% of patients with solar lentigines, with an average clearance of 74.2% of lesions [56]. Treatment with the 1064 nm Q‐Switched (QS) Nd:YAG laser achieved over 50% clearance in 62.5% of solar lentigo patients, with no significant improvement in larger lesions and a recurrence rate of 12.7% [35]. The 532 nm QS Nd:YAG laser significantly outperformed the 1064 nm version in clinical improvement and patient satisfaction for treating solar lentigines [34]. Conversely, both the 532 nm and 660 nm QS Nd:YAG lasers demonstrated significant therapeutic efficacy for solar lentigines. However, the reduction in melanin levels was more pronounced in lesions treated with the 660 nm QS Nd:YAG laser [40]. Clinically excellent improvement in solar lentigines lesions was reported in 93.02% of patients treated with the 532 nm picosecond (PS) Nd:YAG laser. This was accompanied by significant patient satisfaction, a reduction in the melanin index, and notable histological improvement [32]. Additionally, the 532 nm PS Nd:YAG laser demonstrated significantly higher lesion clearance scores (2.95 vs. 1.8) and a lower incidence of PIH (5% vs. 30%) compared to the 532 nm QS Nd:YAG laser [26]. On the other hand, the 532 nm QS KTP laser achieved excellent improvement in pigment clearance in 71.4% of treated lesions, compared to 67.9% for lesions treated with the 532 nm PS KTP laser; this difference was not statistically significant. However, patient satisfaction scores were significantly higher for those treated with the 532 nm PS KTP laser [31]. The 532 nm QS Nd:YAG laser demonstrated efficacy in 76.6% of patients, with 56.6% experiencing good to excellent improvement. In contrast, cryotherapy showed efficacy in 53.3% of patients, with only 43.3% achieving good to excellent improvement. The difference between the two treatments was statistically significant [27]. Similarly, 532 nm QS Nd:YAG laser therapy led to more significant lightening effects and higher patient satisfaction, as well as fewer adverse effects, in the treatment of solar lentigines compared to the other two laser modalities and cryotherapy [1]. The 532 nm QS Nd:YAG laser demonstrated significantly greater pigmentation improvement compared to ablation with fractional CO_2_ laser, achieving over 90% pigment clearance in 36% of patients versus just 8% with fractional CO_2_. Additionally, 80% of patients treated with the QS Nd:YAG laser reported excellent results, compared to only 8% in the fractional CO_2_ group [36]. Treatment with the Er:YAG micropeel effectively reduced pigmentation in solar lentigines lesions. However, the immediate effects of the 532 nm QS Nd:YAG laser were more pronounced than those of the Er:YAG micropeel, achieving over 75% improvement in 26.66% of cases compared to just 6.66% for the micropeel. Nonetheless, a greater degree of PIH occurred following the 532 nm QS Nd:YAG treatment compared to the Er:YAG micropeel [42]. Notably, the degree of pigment reduction following the combined therapy of the Er:YAG micropeel and 532 nm QS Nd:YAG laser was comparable to that of the 532 nm QS Nd:YAG laser alone [43]. The 694 nm QS ruby laser treatment resulted in excellent improvement (> 75%) in 59.34% of patients after the first session of therapy, while complete lesion clearance was achieved in all patients after the second session [48]. Also, QS ruby laser treatment was associated with significant lightening of solar lentigines lesions. It proved superior to topical combinations, offering faster improvement and longer lasting lightening effects, although it also resulted in more side effects [37]. Additionally, the QS ruby laser was significantly more effective than the ablative CO_2_ fractional laser for lesion removal, with over 75% improvement observed in 36.36% of patients treated with the QS ruby laser, compared to only 9.09% of patients with the CO_2_ fractional laser after follow‐up. Also, patient satisfaction was high and moderate for treatment with the QS ruby laser and ablative fractionated CO_2_ laser, respectively [39]. However, a study reported that cryotherapy provided significantly more substantial lightening effects and a higher rate of excellent improvement compared to both argon and CO_2_ laser therapy (37% vs. 25% vs. 23%, respectively) [57] (Table 3).

Chemical peels with 35% trichloroacetic acid (TCA) significantly improved solar lentigines, achieving > 50% lightening in 46% of patients; similar to cryotherapy, which had > 50% lightening in 60% of cases. However, TCA therapy had fewer side effects (13.3%) compared to cryotherapy (40%) [47]. In other studies, cryotherapy was significantly associated with a higher rate of improvement compared to both TCA 33% (> 50% lightening in 40% vs. 12% of patients) [49] and TCA 30% (> 50% lightening in 71.4% vs. 42.8% of patients) [53] (Table 3).

Mild pain and PIH were the most frequent and the most significant adverse effects of laser therapy, cryotherapy, and chemical peels. Also, hypopigmentation, erythema, swelling, skin dryness, pruritus, crusting, burning, and edema were reported. However, most of the adverse effects were mild and transient. Among laser therapy, PDL and IPL modalities seemed to be less likely associated with PIH. No serious adverse effects were reported during the treatment and follow‐up period after laser therapy. However, cryotherapy was mostly associated with more severe side effects. Blisters, severe pain, scarring, permanent erythema, and intense itching were also reported following cryotherapy (Table 3).

## 4 Discussion

This systematic review on clinical trials provides a comprehensive overview of multiple therapeutic modalities for solar lentigines, assessing their efficacy and safety. The findings indicate that, among monotherapies, laser therapy demonstrated greater effectiveness compared to topical medications, cryotherapy, and peels. Furthermore, combination therapy that includes laser treatment along with certain topical agents, as an adjuvant therapy, results in enhanced therapeutic outcomes and a reduction in adverse effects, particularly PIH.

The quality‐switched lasers were used more frequently in the treatment of solar lentigines because they effectively target melanin. This is due to the relatively large size of melanosomes and their quick thermal relaxation time. Consequently, quality‐switched lasers can deliver high‐intensity pulses in the nanosecond range, making them particularly effective for treating conditions such as lentigines [11, 32, 48]. There are various methods of QS laser therapy, including QS Nd:YAG, QS ruby, and QS KTP lasers, all of which have demonstrated notable effects in clearing solar lentigines lesions. Among these, the QS Nd:YAG laser was more frequently studied [31, 34, 35, 40, 48]. However, the PS Nd:YAG laser showed significantly higher efficacy and a lower incidence of PIH compared to the QS Nd:YAG laser [26, 32]. In contrast, the QS KTP laser was associated with more improvement in pigment clearance (though not statistically significant) and greater patient satisfaction (statistically significant) compared to the PS KTP laser [31]. Additionally, QS Nd:YAG [36] and QS ruby [39] laser therapies were linked to greater improvement and higher patient satisfaction compared to fractional CO_2_ laser treatments. However, fractional CO_2_ laser therapy had faster healing times and lower pain scores than the QS Nd:YAG laser [36]. Other laser therapies and energy‐based devices, such as PDL [13, 24, 44] and IPL [29, 45, 56], also demonstrated significant clinical and histological effects on solar lentigines lesions, particularly after two treatment sessions, with a lower likelihood of PIH compared to other laser modalities. Moreover, the QS Nd:YAG laser showed more pronounced effects than the Er:YAG micropeel, but with a higher incidence of PIH [42]. Notably, the pigment reduction from combined therapy (Er:YAG micropeel plus QS Nd:YAG) was similar to using QS Nd:YAG alone, despite a higher PIH rate [43].

Besides, various topical medications are used to treat solar lentigines, often in combination with two or more agents. The combination of mequinol and tretinoin has been extensively evaluated in multiple studies, demonstrating significant therapeutic effects, particularly on facial lesions compared to those on the upper limbs. Notably, some beneficial effects of the treatment persisted even after discontinuation of mequinol and tretinoin, especially on facial lesions [17, 50, 51, 52].

While laser therapy is usually associated with faster improvement and longer‐lasting effects than topical agents, it can also result in more side effects [37]. Therefore, the use of topical medications as adjunct therapies to laser therapy has been demonstrated to enhance therapeutic outcomes and reduce the risk of post‐inflammatory hyperpigmentation (PIH), a common concern among patients undergoing laser treatment [25]. This synergistic effect could be particularly beneficial for patients who have experienced previous unsuccessful treatments or those with resistant pigmentation. The reduced incidence of PIH with combination therapies is particularly noteworthy, as this complication often dissuades patients from pursuing treatment for solar lentigines, especially in individuals with darker skin types.

Also, laser therapy with QS Nd:YAG laser [1, 27] and PDL [13] therapy demonstrated more significant therapeutic effects and higher patient satisfaction, as well as fewer adverse effects, in the treatment of solar lentigines compared to cryotherapy [1], even for skin types III and IV. However, cryotherapy showed more improvement compared to both argon and CO_2_ laser therapy [57]. Treatment of solar lentigines lesions with topical agents after ablative cryotherapy could enhance the treatment outcomes of cryotherapy [19, 38]. Chemical peels with trichloroacetic acid (TCA) have demonstrated significant results in treating solar lentigines. In higher concentrations, TCA is as effective as cryotherapy [47], while in lower concentrations, it may be less effective than cryotherapy [49, 53]. Notably, TCA therapy is associated with fewer side effects and a shorter healing time compared to cryotherapy [47, 49, 53].

In cryotherapy, the mechanism involves the localized freezing of the affected skin areas, leading to the destruction of melanocytes [58]. Melanocytes are particularly susceptible to damage from cold temperatures, with injuries occurring at temperatures ranging from −4°C to −7°C [59]. This controlled freeze–thaw cycle results in cellular damage, inducing a natural inflammatory response and subsequent peeling or sloughing of the treated skin [60]. On the other hand, TCA acts as a chemical exfoliant by penetrating the epidermis and dermis, leading to the controlled coagulation of proteins in the skin. This process stimulates collagen synthesis and promotes the regeneration of new skin layers, resulting in improved texture and tone [61, 62]. The depth and extent of the peel can be adjusted based on concentration and application time [63].

A systematic review of treatments for lentigines found that combination‐based therapies were the most efficacious therapeutic approach for solar lentigines (complete responses of 65%), followed by laser treatments (complete responses 43%) [20]. In this study, which utilized the results of clinical trials, we suggest that laser therapy is the most effective treatment for solar lentigines, especially when combined with certain topical agents as an adjuvant therapy.

This systematic review presents several limitations that should be acknowledged. One of them is the potential heterogeneity among the included studies. Variations in study design, treatment protocols, participant demographics, and outcome measurements can significantly impact the generalizability of the findings. Additionally, many trials may exhibit methodological limitations, such as small sample sizes or short follow‐up durations, which constrain the reliability of the efficacy conclusions drawn. Furthermore, publication bias may also skew the results, as studies with positive outcomes are more likely to be published than those with negative or inconclusive results. This selection bias can lead to an overestimation of treatment effectiveness. Lastly, the lack of standardized definitions and assessment tools for solar lentigines across the literature complicates the comparison of treatment outcomes, ultimately hindering the development of unified treatment guidelines. Also, most of the clinical trials included in this review primarily compared two treatment modalities. However, the number of randomized controlled trials featuring a control group was limited. Additionally, the lack of raw data, along with the diversity in outcome measures, precluded the possibility of conducting a meta‐analysis. These factors hinder our ability to draw more definitive conclusions regarding the relative efficacy of the various treatments for solar lentigines.

In conclusion, the available findings suggest that laser therapy may offer greater effectiveness compared to other therapeutics, with a well‐tolerated safety profile. Additionally, combining laser therapy with specific topical agents could enhance therapeutic outcomes and reduce PIH. However, further large‐scale randomized clinical trials are necessary to validate these results.

## Author Contributions

Fahimeh Abdollahimajd, Mohammad Javad Nasiri, and Nastaran Namazi conceptualized and designed the methodology for this study. Mohammad Javad Nasiri and Ghazal Mardani undertook the systematic literature search and critical appraisal. Ghazal Mardani prepared the original draft. Fahimeh Abdollahimajd, Mohammad Javad Nasiri, Mehdi Farshchian, and Nastaran Namazi critically revised the manuscript. All authors contributed to the critical review, commentary, and revision of the original manuscript.

## Conflicts of Interest

The authors declare no conflicts of interest.

## Supporting information


Appendix S1

## Data Availability

Data sharing is not applicable to this article as no new datasets were generated or analyzed during the current study. The study protocol and detailed search strategy were registered in PROSPERO(CRD42024574497).
