# Supplementary material for: Treatment of Solar Lentigines: A Systematic Review of Clinical Trials
Source: J Cosmet Dermatol. 2025 Mar 27;24(4):e70133. doi: 10.1111/jocd.70133 (PMC11948172; doi:10.1111/jocd.70133)
Supplement: Supplementary file 1 — Appendix S1 [file JOCD-24-e70133-s001.docx]

**Tables’ References**

1. Makino ET, Huang P, Cheng T, Acevedo SF, de Oliveira C, Mehta RC. 12-week, single-center study of a targeted pigment-correcting dark spot treatment for post-inflammatory hyperpigmentation and solar lentigines. Clinical, Cosmetic and Investigational Dermatology. 2023:2677-86.
2. Kim HO, Kim HR, Kim JC, Kang SY, Jung MJ, Chang SE, et al. A randomized controlled trial on the effectiveness of epidermal growth factor-containing ointment on the treatment of solar lentigines as adjuvant therapy. Medicina. 2021;57(2):166.
3. Ishikawa Y, Niwano T, Hirano S, Numano K, Takasima K, Imokawa G. Whitening effect of L-ascorbate-2-phosphate trisodium salt on solar lentigos. Archives of Dermatological Research. 2019;311:183-91.
4. Arginelli F, Greco M, Ciardo S, Josse G, Rossi AB, Le Digabel J, et al. Efficacy of D-pigment dermocosmetic lightening product for solar lentigo lesions of the hand: A randomized controlled trial. Plos one. 2019;14(5):e0214714.
5. Jiang L, Hino PD, Bhatia A, Stephens TJ, Jimenez F. Efficacy of Trifecting® night cream, a novel triple acting skin brightening product: A double-blind, placebo-controlled clinical study. The Journal of Clinical and Aesthetic Dermatology. 2018;11(12):21.
6. Campanati A, Giannoni M, Scalise A, De Blasio S, Giuliano A, Giuliodori K, et al. Efficacy and safety of topical pidobenzone 4% as adjuvant treatment for solar lentigines: result of a randomized, controlled, clinical trial. Dermatology. 2016;232(4):478-83.
7. Morag M, Nawrot J, Siatkowski I, Adamski Z, Fedorowicz T, Dawid‐Pac R, et al. A double‐blind, placebo‐controlled randomized trial of Serratulae quinquefoliae folium, a new source of β‐arbutin, in selected skin hyperpigmentations. Journal of Cosmetic Dermatology. 2015;14(3):185-90.
8. Hexsel D, Hexsel C, Porto M, Siega C. Triple combination as adjuvant to cryotherapy in the treatment of solar lentigines: investigator‐blinded, randomized clinical trial. Journal of the European Academy of Dermatology and Venereology. 2015;29(1):128-33.
9. Khemis A, Cabou J, Dubois J, Ortonne JP. A randomized controlled study to evaluate the depigmenting activity of l‐ascorbic acid plus phytic acid–serum vs. placebo on solar lentigines. Journal of cosmetic dermatology. 2011;10(4):266-72.
10. Katoulis A, Alevizou A, Bozi E, Makris M, Zafeiraki A, Mantas N, et al. A randomized, double‐blind, vehicle‐controlled study of a preparation containing undecylenoyl phenylalanine 2% in the treatment of solar lentigines. Clinical and experimental dermatology. 2010;35(5):473-6.
11. Jarratt M. Mequinol 2%/tretinoin 0.01% solution: an effective and safe alternative to hydroquinone 3% in the treatment of solar lentigines. Cutis. 2004;74(5):319-22.
12. Draelos ZD. The combination of 2% 4‐hydroxyanisole (mequinol) and 0.01% tretinoin effectively improves the appearance of solar lentigines in ethnic groups. Journal of Cosmetic Dermatology. 2006;5(3):239-44.
13. Ortonne JP, Camacho F, Wainwright N, Bergfelt L, Westerhof W, Roseeuw D. Safety and efficacy of combined use of 4-hydroxyanisole (mequinol) 2%/tretinoin 0.01% solution and sunscreen in solar lentigines. Cutis. 2004;74(4):261-4.
14. Kang S, Goldfarb MT, Weiss JS, Metz RD, Hamilton TA, Voorhees JJ, et al. Assessment of adapalene gel for the treatment of actinic keratoses and lentigines: a randomized trial. Journal of the American Academy of Dermatology. 2003;49(1):83-90.
15. Hermanns J-F, Petit L, Piérard-Franchimont C, Paquet P, Piérard G. Assessment of topical hypopigmenting agents on solar lentigines of Asian women. Dermatology. 2002;204(4):281-6.
16. Fleischer Jr AB, Schwartzel EH, Colby SI, Altman DJ. The combination of 2% 4-hydroxyanisole (Mequinol) and 0.01% tretinoin is effective in improving the appearance of solar lentigines and related hyperpigmented lesions in two double-blind multicenter clinical studies. Journal of the American Academy of Dermatology. 2000;42(3):459-67.
17. Abd. El-Naby N, Mostafa Ali M, Hawwam SA, Sarhan N. The clinical and electron microscopic evaluation of the impact of pulsed dye laser techniques on solar lentigines (randomized clinical trial). Journal of Dermatological Treatment. 2022;33(1):361-8.
18. Kim JY, Yang J, Huh G, Choi Y-J, Kim W-S. A split-face, single-blinded, randomized controlled comparison of 532 nm picosecond neodymium-doped yttrium aluminum garnet laser versus 532 nm Q-switched neodymium-doped yttrium aluminum garnet laser in the treatment of solar lentigines. Annals of Dermatology. 2020;32(1):8.
19. Dawood N, Tahir K, Shahid M, Aman S, Jawaid K, Ali U. Comparison of efficacy of cryotherapy vs Q-switch Nd: YAG Laser in the treatment of solar lentigines. Journal of Pakistan Association of Dermatologists. 2020;30(2):277-81.
20. Friedmann DP, Peterson JD. Efficacy and safety of intense pulsed light with a KTP filter for the treatment of solar lentigines. Lasers in Surgery and Medicine. 2019;51(6):500-8.
21. Vachiramon V, Iamsumang W, Triyangkulsri K. Q-switched double frequency Nd: YAG 532-nm nanosecond laser vs. double frequency Nd: YAG 532-nm picosecond laser for the treatment of solar lentigines in Asians. Lasers in Medical Science. 2018;33:1941-7.
22. Negishi K, Akita H, Matsunaga Y. Prospective study of removing solar lentigines in Asians using a novel dual‐wavelength and dual‐pulse width picosecond laser. Lasers in surgery and medicine. 2018;50(8):851-8.
23. Bohnert K, Dorizas A, Sadick N. A prospective, randomized, double-blinded, split-face pilot study comparing Q-switched 1064-nm Nd: YAG versus 532-nm Nd: YAG laser for the treatment of solar lentigines. Journal of Cosmetic and Laser Therapy. 2018;20(7-8):395-7.
24. Kaminaka C, Furukawa F, Yamamoto Y. The clinical and histological effect of a low-fluence Q-switched 1,064-nm neodymium: yttrium-aluminum-garnet laser for the treatment of melasma and solar lentigenes in Asians: prospective, randomized, and split-face comparative study. Dermatologic Surgery. 2017;43(9):1120-33.
25. Vachiramon V, Panmanee W, Techapichetvanich T, Chanprapaph K. Comparison of Q‐switched Nd: YAG laser and fractional carbon dioxide laser for the treatment of solar lentigines in Asians. Lasers in Surgery and Medicine. 2016;48(4):354-9.
26. Imhof L, Dummer R, Dreier J, Kolm I, Barysch MJ. A prospective trial comparing q-switched ruby laser and a triple combination skin-lightening cream in the treatment of solar lentigines. Dermatologic Surgery. 2016;42(7):853-7.
27. Schoenewolf NL, Hafner J, Dummer R, Bogdan Allemann I. Laser treatment of solar lentigines on dorsum of hands: QS Ruby laser versus ablative CO 2 fractional laser–a randomized controlled trial. European Journal of Dermatology. 2015;25:122-6.
28. Noh TK, Chung BY, Yeo UC, Chang S, Lee MW, Chang SE. Q-Switched 660-nm versus 532-nm Nd: YAG laser for the treatment for facial lentigines in Asian patients: a prospective, randomized, double-blinded, split-face comparison pilot study. Dermatologic Surgery. 2015;41(12):1389-95.
29. Jun HJ, Kim SM, Choi WJ, Cho SH, Lee JD, Kim HS. A split-face, evaluator-blind randomized study on the early effects of Q-switched Nd: YAG laser versus Er: YAG micropeel in light solar lentigines in Asians. Journal of Cosmetic and Laser Therapy. 2014;16(2):83-8.
30. Jun HJ, Cho SH, Lee JD, Kim HS. A split-face, evaluator-blind randomized study on the early effects of Q-switched Nd: YAG laser plus Er: YAG micropeel (combined therapy) versus Q-switched Nd: YAG alone in light solar lentigines in Asians. Lasers in Medical Science. 2014;29:1153-8.
31. Ghaninejhadi H, Ehsani A, Edrisi L, Gholamali F, Akbari Z, Noormohammadpour P. Solar lentigines: Evaluating pulsed dye laser (PDL) as an effective treatment option. Journal of Lasers in Medical Sciences. 2013;4(1):33.
32. Seirafi H, Fateh S, Farnaghi F, Ehsani AH, Noormohammadpour P. Efficacy and safety of long-pulse pulsed dye laser delivered with compression versus cryotherapy for treatment of solar lentigines. Indian Journal of Dermatology. 2011;56(1):48-51.
33. Sasaya H, Kawada A, Wada T, Hirao A, Oiso N. Clinical effectiveness of intense pulsed light therapy for solar lentigines of the hands. Dermatologic Therapy. 2011;24(6):584-6.
34. Golforoushan F, Azimi H, SADEGHI M, Yousefi N, GOLDOUST M. Efficacy and side effects of trichloroacetic acid (TCA) versus cryotherapy in the treatment of solar lentigo. Iranian Journal of Dermatology. 2010;13:47-50.
35. Sadighha A, Saatee S, Muhaghegh-Zahed G. Efficacy and adverse effects of Q-switched ruby laser on solar lentigines: a prospective study of 91 patients with Fitzpatrick skin type II, III, and IV.Dermatologic surgery. 2008;34(11):1465-8.
36. Raziee M, Balighi K, Shabanzadeh‐Dehkordi H, Robati R. Efficacy and safety of cryotherapy vs. trichloroacetic acid in the treatment of solar lentigo. Journal of the European Academy of Dermatology and Venereology. 2008;22(3):316-9.
37. Lugo‐Janer A, Lugo‐Somolinos A, Sanchez JL. Comparison of trichloroacetic acid solution and cryosurgery in the treatment of solar lentigines. International journal of dermatology. 2003;42(10):829-31.
38. Todd MM, Rallis TM, Gerwels JW, Hata TR. A comparison of 3 lasers and liquid nitrogen in the treatment of solar lentigines: a randomized, controlled, comparative trial. Archives of dermatology. 2000;136(7):841-6.
39. Bjerring P, Christiansen K. Intense pulsed light source for treatment of small melanocytic nevi and solar lentigines. Journal of Cutaneous Laser Therapy. 2000;2(4):177-81.
40. Hexsel DM, Mazzuco R, Bohn J, Borges J, Gobbato DO. Clinical comparative study between cryotherapy and local dermabrasion for the treatment of solar lentigo on the back of the hands. Dermatologic surgery. 2000;26(5):457-62.
41. Stem RS, Dover JS, Levin JA, Arndt KA. Laser therapy versus cryotherapy of lentigines: a comparative trial. Journal of the American Academy of Dermatology. 1994;30(6):985.
